# Development of a Real‐Time PCR Assay for Noninvasive Detection of *Miamiensis avidus* in Olive Flounder Aquaculture

**DOI:** 10.1111/jfd.14134

**Published:** 2025-04-23

**Authors:** Jin‐Young Kim, Su‐Mi Shin, Se Ryun Kwon, Sung‐Ju Jung

**Affiliations:** ^1^ Department of Aqualife Medicine Chonnam National University Yeosu Republic of Korea; ^2^ Department of Aquatic Life Medical Sciences Sunmoon University Asan Republic of Korea

**Keywords:** cytochrome c oxidase subunit I (*cox1*), *Miamiensis avidus*, noninvasive, *Philasterides dicentrarchi*, real‐time PCR

## Abstract

*Miamiensis avidus* (syn. *Philasterides dicentrarchi*) leads to high mortality and economic losses in olive flounder ( *Paralichthys olivaceus* ) aquaculture. In this study, we developed a real‐time PCR assay targeting the cytochrome c oxidase subunit I (*cox1*) gene of *M*. *avidus* to sensitively detect and quantify the parasite in seawater. The assay showed a strong linear correlation between the log copy number of the standard plasmid and Ct values (*R* ^2^ = 0.9985), achieving an amplification efficiency of 96.26%. The *cox1* gene copy number was estimated at 6017 ± 2794 copies per cell. The assay specifically detected *M*. *avidus* (*cox1* genotypes 1–4) without cross‐reacting with *Cryptocaryon irritans* or *Uronema elegans* , identifying as few as two *M*. *avidus* cells in 1 L of seawater with 100% accuracy (Ct value: 31.95 ± 0.78). The noninvasive seawater sampling demonstrates higher sensitivity than invasive tissue sampling. A high density of *M*. *avidus* (57.81 cells/10 L) was detected in tanks housing olive flounder with clinical signs of infection, and sporadic detection occurred in both inflow and effluent seawater. The developed real‐time PCR assay provides a sensitive, specific and noninvasive method for detecting and quantifying *M*. *avidus*, enabling improved disease prevention and management in fish farms.

## 1 Introduction

Scuticociliatosis, caused by ciliated protozoa of the class Oligohymenophorea and subclass Scuticociliatia, is one of the most serious diseases impacting marine aquaculture worldwide (Budiño et al. 2011; Feng et al. 2015; Jung et al. 2005; Kim et al. 2022; Shin et al. 2021). In Korea, four species of scuticociliates have been identified: *Uronema marinum*, *Miamiensis avidus* (syn. *Philasterides dicentrarchi*), *Pseudocohnilembus persalinus* and *Pseudocohnilembus hargisi*, with *M*. *avidus* being the most pathogenic to olive flounder ( *Paralichthys olivaceus* ) (Jung and Jung 2021; Lee et al. 2018; Song et al. 2009; Whang et al. 2013). This parasite quickly invades the olive flounder through the gills and skin, causing severe systemic infections and high mortality, resulting in significant economic losses in the aquaculture industry (Jung et al. 2011a; Shin et al. 2021, 2023). While infection occurs at all ages, younger fish exhibit a higher mortality rate at higher temperatures. To mitigate these impacts, regular monitoring of fish farms, along with improved handling practices and environmental management, is essential for controlling the spread of this disease.

Current detection methods for *M*. *avidus* involve microscopic examination of infected tissues or polymerase chain reaction (PCR)–based nucleic acid detection (Bae et al. 2009; Jung et al. 2005; Whang et al. 2013). However, these methods often require sacrificing the fish, as they involve removing fish from the culture tank and excising gill and skin tissues for examination. Moreover, by the time clinical signs appear, the infection has often spread throughout the farm. To overcome these limitations, recent studies have demonstrated that DNA from water samples can be used for noninvasive detection of aquatic pathogens, including *Aeromonas salmonicida* in rainbow trout and *Bonamia ostreae* in oysters before clinical signs appear (Jørgensen et al. 2020; Marana et al. 2024). Additionally, highly sensitive real‐time PCR‐based methods have been developed for detecting bacterial pathogens such as *Yersinia ruckeri* in fish faeces, providing a reliable and nonlethal diagnostic alternative (Ghosh et al. 2018). These approaches highlight the potential of combining noninvasive sampling with highly sensitive PCR techniques for early pathogen detection, enabling disease monitoring and intervention without sacrificing fish.

Real‐time PCR has become a critical tool for estimating parasite loads and monitoring infection progression in aquaculture (Agawa et al. 2016; Alonso et al. 2015; Bridle et al. 2010; Collins et al. 2010; Griffin et al. 2008; Ishimaru et al. 2014; Piazzon et al. 2012; Shin et al. 2019; Sueiro et al. 2022; Tang et al. 2022; Taniguchi et al. 2011). Several studies have utilised real‐time PCR to detect *M*. *avidus* by targeting genes such as 18S rDNA, nuclear large subunit ribosomal DNA (LSU rDNA) and the internal transcribed spacer 2 (ITS‐2) region of the nuclear genome (Kim et al. 2019; Power et al. 2019; Sueiro et al. 2022). However, due to the abundance of ciliate species in natural seawater, nuclear genome‐based sequences may not provide sufficient specificity for species‐level detection.

To address this limitation, we focused on the mitochondrial genome, specifically the cytochrome c oxidase subunit I (*cox1*) gene, which displays high variability and has been widely used to study intraspecific genetic variation in various ciliate species (Budiño et al. 2011; Jung et al. 2011b; Whang et al. 2013). In this study, we developed a real‐time PCR assay targeting the *cox1* gene of *M*. *avidus* for noninvasive detection in olive flounder farms. By using noninvasive sampling methods, this assay provides highly specific and sensitive detection of *M*. *avidus*, enhancing monitoring and disease control efforts in aquaculture.

## 2 Materials and Methods

### 2.1 Ethics Statement

The animal experiment procedures in this study were performed following the guidelines and permits approved by the Institutional Animal Care and Use Committee (IACUC) of Chonnam National University (CNU IACUC‐YS‐2021‐3).

### 2.2 Ciliate Isolation and Culture

*M*. *avidus* was isolated from infected olive flounder as previously described and identified through analysis of the 18S rDNA and mitochondrial *cox1* gene (Jung et al. 2011b). *M*. *avidus* was maintained and subcultured at intervals of 1–2 months at 10°C in the Chinook salmon embryo (CHSE)‐214 cell line, cultured in Dulbecco's Modified Eagle Medium (DMEM; Gibco, USA) supplemented with 50 IU/mL penicillin G, 50 μg/mL streptomycin (Gibco, USA) and 10% fetal bovine serum (FBS; Gibco, USA). For the experiment, the incubation temperature was raised to 20°C, with subcultures carried out every 5–7 days. The *M*. *avidus* strains used in the experiments are listed in Table 1.

**TABLE 1:** *M*. *avidus* isolates used in this study.

*M. avidus* strain	Sampling year	Geographic origin	Host species	GenBank accession	*Cox1* genotype	References
YS2	2005	Korea, Yosu	*P. olivaceus*	EU831221	1	Jung et al. 2011b
JJB	2014	Korea, Jeju	*P. olivaceus*	—	1	Sohn et al. 2023
SJF‐03B	2003	Korea, Wando	*P. olivaceus*	EU831216	2	Jung et al. 2011b
A3	2006	Korea, Jeju	*P. olivaceus*	EU831214	3	Jung et al. 2011b
Mie0301	2003	Japan, Owase	*P. olivaceus*	EU831233	4	Jung et al. 2011b
BB19	2022	Korea, Jeju	*P. olivaceus*	—	4	—

*Uronema elegans* was isolated from rotifers ( *Brachionus plicatilis* ), a food source for olive flounder larvae, at an olive flounder farm in Jeju Island, Korea, and identified through 18S rDNA analysis (Tytgat et al. 2019). *U. elegans* was maintained in DMEM supplemented with 50 IU/mL penicillin G, 50 μg/mL streptomycin and 10% FBS at 20°C.

*Cryptocaryon irritans* was isolated from infected olive flounder at a farm in Jeju Island and identified by microscopic observation and PCR using specific primers (Xie et al. 2021). *C. irritans* was maintained by cohabitation infection with 20 olive flounder (15.11 ± 1.83 g) in a 300 L circulating water tank at a water temperature of 20°C. To sustain the infection, dead fish were replaced with an equal number of healthy fish throughout the experiment.

### 2.3 DNA Extraction

DNA was extracted from *M*. *avidus*, *C. irritans* , *U. elegans* , fertilised eggs and olive flounder fingerlings (30, 60 and 90 days old) using the QIAamp DNA Mini Kit (QIAGEN, Germany) according to the manufacturer's instructions. The extracted DNA was eluted in 100 μL (two aliquots of 50 μL) and stored at −80°C until use.

Filtered seawater membrane samples (prepared as described in Sections 2.8 and 2.9) were transferred to a bead tube (Innogenetech, Korea) containing 300 μL of ATL buffer (QIAGEN, Germany) and disrupted for 30 s at 4000 rpm using a BeadBug microtube homogeniser (Benchmark Scientific, USA). DNA was then extracted using the QIAamp DNA Mini Kit (QIAGEN, Germany), with the reagent volumes increased 1.5‐fold. The extracted DNA was eluted in 100 μL (two aliquots of 50 μL) and stored at −80°C until use.

### 2.4 Primer Design for Real‐Time PCR Assay

Real‐time PCR primers were designed using the Primer3Plus program and Primer‐BLAST analysis, targeting the 159–274 bp region of the *cox1* gene to ensure the detection of all known *cox1* genotypes of *M. avidus* (Genotypes 1–4) (Jung et al. 2011b) (Tables 1 and 2). The *cox1* sequences of other ciliates, including *Uronema* spp., *Pseudocohnilembus* spp., *Trichodina* spp., *Metanophrys* spp., *Tetrahymena* spp., *Ichthyophthirius* spp. and *Cryptocaryon* spp. deposited in the NCBI database (Table S1), had at least six sequence mismatches with the designed primers. The primer design criteria included a length of 15–25 bp, GC content of 30%–60% and an amplification size of 100–200 bp, while minimising the formation of primer dimers and hairpin loops. Gradient PCR was performed using an AllInOneCycler (Bioneer, Korea) to determine the optimal annealing temperature for the designed primers.

**TABLE 2:** Primers used for the development of the *M. avidus* real‐time PCR assay in this study.

Target		Primer sequences (5′‐3′)	Use	References
*M*. *avidus* cox1	F199d‐B2	TCAGGAGCTGCMTTAGCHACYATG	Gene cloning	Jung et al. 2011b
	R1143d	TARTAGGATCMCCWCCATAAGC		
	*M*. *avidus* qF	TGGTTCTAAAGATGTGGCTTACC	Real‐time PCR	In this study
	*M*. *avidus* qR	AGTATCTTCAGTATTGTTGGCCTT		

### 2.5 Real‐Time PCR Assay

Real‐time PCR was performed using the Exicycler 96 Real‐Time PCR system (Bioneer, Korea). The reaction was carried out in a final volume of 20 μL, consisting of 10 μL of AccuPower Greenstar qPCR Premix (SYBR Green; Bioneer, Korea), 1 μL of DNA template, 0.5 μL (5 pM) of each primer and 8 μL of diethylpyrocarbonate (DEPC)‐treated water (Invitrogen, USA). Thermal cycling conditions were as follows: an initial denaturation at 95°C for 10 min, followed by 40 cycles of denaturation at 95°C for 20 s and annealing/extension at 58°C for 40 s. The cycle threshold (Ct) value was determined using the automatic settings of the Exicycler 96 Real‐Time PCR system. After the PCR, amplification specificity was confirmed through melting curve analysis and agarose gel electrophoresis. All real‐time PCR reactions were performed in triplicate.

### 2.6 Cloning and Copy Number Determination

The *cox1* gene of *M*. *avidus* (strain: YS2; Table 1) was amplified using universal *cox1* gene primers (Table 2). The PCR products were cloned into the pGEM‐T Easy vector (Promega, USA) and transformed into DH5α Chemically Competent *E. coli* (Enzynomics, Korea). Plasmids from the selected transformant clones were extracted using the AccuPrep Nano‐Plus Plasmid Extraction Kit (Bioneer, Korea), and the concentration was measured using a NanoPhotometer N60 (Implen, Germany). The plasmid copy number was calculated using the following equation: (amount × 6.022 × 10^23^)/(length × 1 × 10^9^ × 650). A standard curve was prepared by plotting Ct values against plasmid copy numbers serially diluted 10‐fold.

The threshold was set at 95% of the limit of detection (LoD_95%_). After serially diluting the plasmid by 2‐fold (400, 200, 100, 50, 25, 12.5, 6.25, 3.125, 1.5625, 0.78125 copies/μL/reaction), each dilution was analysed in 24 replicates (triplicates per reaction). The results were analysed by probit regression using SPSS statistical software (SPSS Inc., USA) to determine the LoD_95%_.

### 2.7 Estimation of cox1 Gene Copy Number in *M. Avidus*

To determine the *cox1* gene copy number in a single *M*. *avidus* cell, the YS2 strain was counted using a haemocytometer. A concentration of 4 × 10^6^ cells/mL was serially diluted 10‐fold, and the cells were counted at 400,000, 40,000, 4000, 400, 40 and 4 cells per 100 μL. All samples were stored at −80°C until DNA extraction. The experiments were performed in triplicate. The total copy number of the *cox1* gene in *M*. *avidus* was calculated using the following formula: Total *cox1* gene copy number = copy number in 1 μL template × elution volume (100).

### 2.8 Specificity Assessment

The specificity of the primers was evaluated using five strains of *M*. *avidus* (*cox1* genotypes 1–4; Table 1), as well as *U. elegans* and *C. irritans* . A total of 1 × 10^3^ cells of *M*. *avidus* and *U. elegans* , and 30 theront‐stage cells of *C. irritans* , were prepared. All samples were stored at −80°C until DNA extraction. The experiment was performed in triplicate. The *cox1* gene copy number per cell was calculated using the following formula: *cox1* gene copy number/cell = {copy number for 1 μL template × elution volume (100)}/total cell number. The results were analysed by one‐way analysis of variance (ANOVA) using SPSS statistical software followed by Tukey's honestly significant difference (HSD) post hoc test. A *p* value less than 0.05 was considered statistically significant.

### 2.9 Sensitivity and Reproducibility Assessment

The sensitivity of the assay was determined by identifying the minimum detectable number of *M*. *avidus* cells in 1 L of seawater, while reproducibility was assessed through 10 repeated experiments. Sand‐filtered inflow seawater, collected from the Maritime and Fisheries Science Museum in Yeosu, Korea, was used to dilute the ciliate at a water temperature of 20°C. Since *M. avidus* cultured in cell culture medium exhibit reduced motility and partial disruption due to osmotic shock after being introduced to seawater, they were acclimated in seawater for 2 h before use in the experiment. Cultured *M*. *avidus* cells were serially diluted in a 96‐well plate (SPL Life Sciences, Korea) using seawater, counted under a microscope at concentrations of 0, 1, 2, 4 and 8 cells, and then added to 1 L of seawater. Each 1‐L seawater sample containing *M*. *avidus* was vacuum filtered through a 5.0 μm pore size mixed cellulose ester (MCE) membrane filter (Merck, Germany), and the filters were immediately stored at −80°C until DNA extraction. The experiment was performed with 10 replicates.

### 2.10 Sample Collection for Monitoring *M. Avidus*

#### 2.10.1 Fish and Rearing Water Sampling Across Developmental Stages

Detection of *M*. *avidus* was carried out using both invasive and noninvasive methods at an olive flounder farm in Jeju Island, Korea, between March 2022 and April 2023. Sampling was performed approximately once a month over three production cycles. During the seed production cycles, the rearing seawater conditions for the broodstock and their progeny were maintained at a water temperature of 18°C–20°C, a pH of 7.8–8.0 and a salinity of 29%–30‰. Fertilised eggs produced at the same facility (200 eggs per 200 μL) and 10 olive flounder fingerlings from age groups of 30 days (0.49 ± 0.05 g), 60 days (1.05 ± 0.37 g) and 90 days (10.77 ± 1.31 g) were collected. For DNA extraction, 200 eggs were used, while whole fingerlings were sampled from the 30‐day‐old group. The 60‐day‐old group samples included gills and pooled skin mucus with fins (dorsal, caudal, anal), and the 90‐day‐old group samples included gills, skin mucus, fins and brain. All samples were stored at −80°C until DNA extraction. Additionally, 10 L of rearing tank seawater was collected for each corresponding developmental stage (fertilised eggs and 30, 60 and 90‐day‐old fish) in sterile collection bottles. The seawater samples were immediately gravity‐filtered through a 250‐μm pore size stainless steel mesh and then vacuum‐filtered through a 5.0 μm pore size MCE membrane filter. Each filtered sample was stored at −80°C until DNA extraction.

### 2.11 Rearing Water Sampling in Diseased, Recovered and Healthy Olive Flounder Tanks

We next sought to quantify the number of *M*. *avidus* cells detected in rearing water during fish mortalities and to assess whether *M*. *avidus* could be detected in recovered fish tanks without clinical signs. Briefly, a tank containing olive flounder infected with *M*. *avidus*, where an average of three fish out of approximately 3000 died daily over the course of a month, was selected. Another tank, deemed a recovery tank, was selected based on the absence of deaths and clinical signs for a month following a period of mild mortality due to *M*. *avidus* infection. A control tank with no history of *M*. *avidus* infection was also selected. For each tank, 10 L of rearing tank seawater was collected and analysed using the real‐time PCR assay.

### 2.12 Sampling of Inflow Natural Seawater Into Farm and Effluent Rearing Water

To assess the presence of *M*. *avidus* in the inflow and effluent water of the olive flounder farm, natural seawater entering the farm and rearing water leaving the farm were sampled. Inflow natural seawater was collected at the farm's entry point, while effluent rearing water was sampled from the sedimentation tank just before its discharge into the sea. The sedimentation tank gathered effluent water from both the seed production facility and the culture farm, where fish were grown until harvest. For each sampling event, 10 L of inflow seawater and 10 L of effluent water were collected in sterile bottles and analysed using the real‐time PCR assay.

Sampling was conducted at approximately 1‐month intervals between March 2022 and April 2023, synchronised with fish and rearing water sampling, to monitor potential contamination from natural seawater and to assess the release of *M*. *avidus* through the effluent water.

## 3 Results

### 3.1 Standard Curve for Quantitative Detection of *M. Avidus*

**FIGURE 1:**
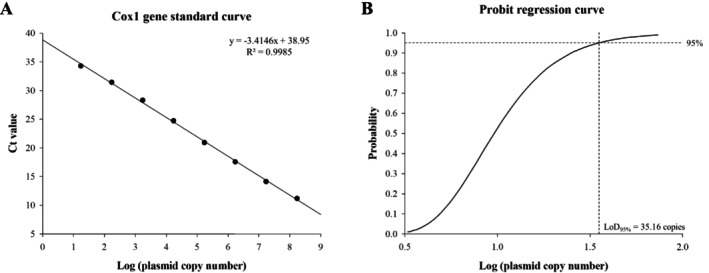
Standard curve of the real‐time PCR for the *cox1* gene of *M. avidus*. A 10‐fold serial dilution of the *cox1* gene‐positive plasmid was used for analysis. Data points represent the average of triplicates. B. The limit of detection (LoD_95%_) was determined by probit regression analysis based on 24 replicates of two‐fold serial dilutions of the positive plasmid.

The correlation between the log copy number and Ct values of the standard plasmid exhibited strong linearity, with an *R* ^2^ value of 0.9985, a calculated slope of −3.4146 and an amplification efficiency of 96.26% (Figure 1A). The LoD_95%_ value was 35.16 copies (95% CI: 18.75–510.51 copies) (Figure 1B), which corresponds to a Ct value of 33.67. Consequently, a Ct value of 33.67 or less was considered positive for detection.

### 3.2 Cox1 Gene Copy Number in *M. Avidus*

**FIGURE 2:**
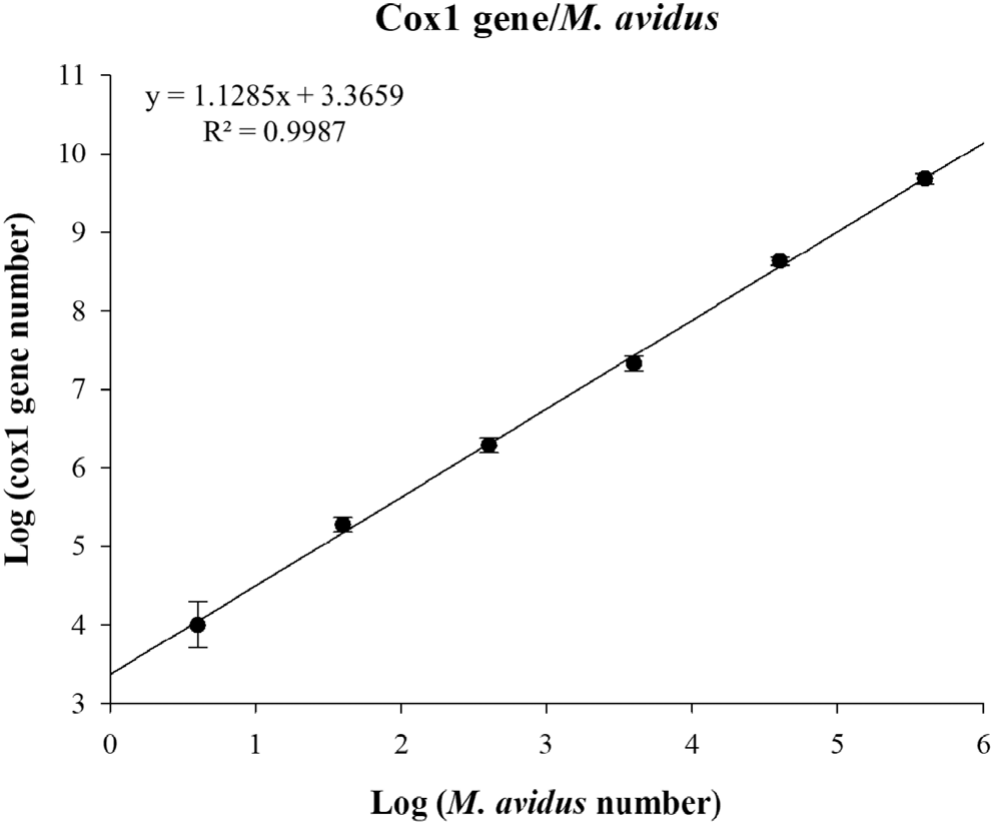
Standard curve showing the relationship between the *cox1* gene copy number and the number of *M. avidus* cells. The *cox1* gene copy numbers were measured in 400,000, 40,000, 4000, 400, 40 and 4 cells (*n* = 3). Data points are expressed as mean ± standard error of the mean.

The correlation between the *cox1* gene copy number and the counted cell number of *M*. *avidus* also showed good linearity, with an R^2^ value of 0.9985 (Figure 2). Based on regression analysis, the *cox1* gene copy number per *M*. *avidus* single cell was estimated at 6017 ± 2794 copies. This value was applied to the *cox1* copy number standard curve to estimate the number of *M*. *avidus* cells in unknown samples.

### 3.3 Specificity Analysis

**FIGURE 3:**
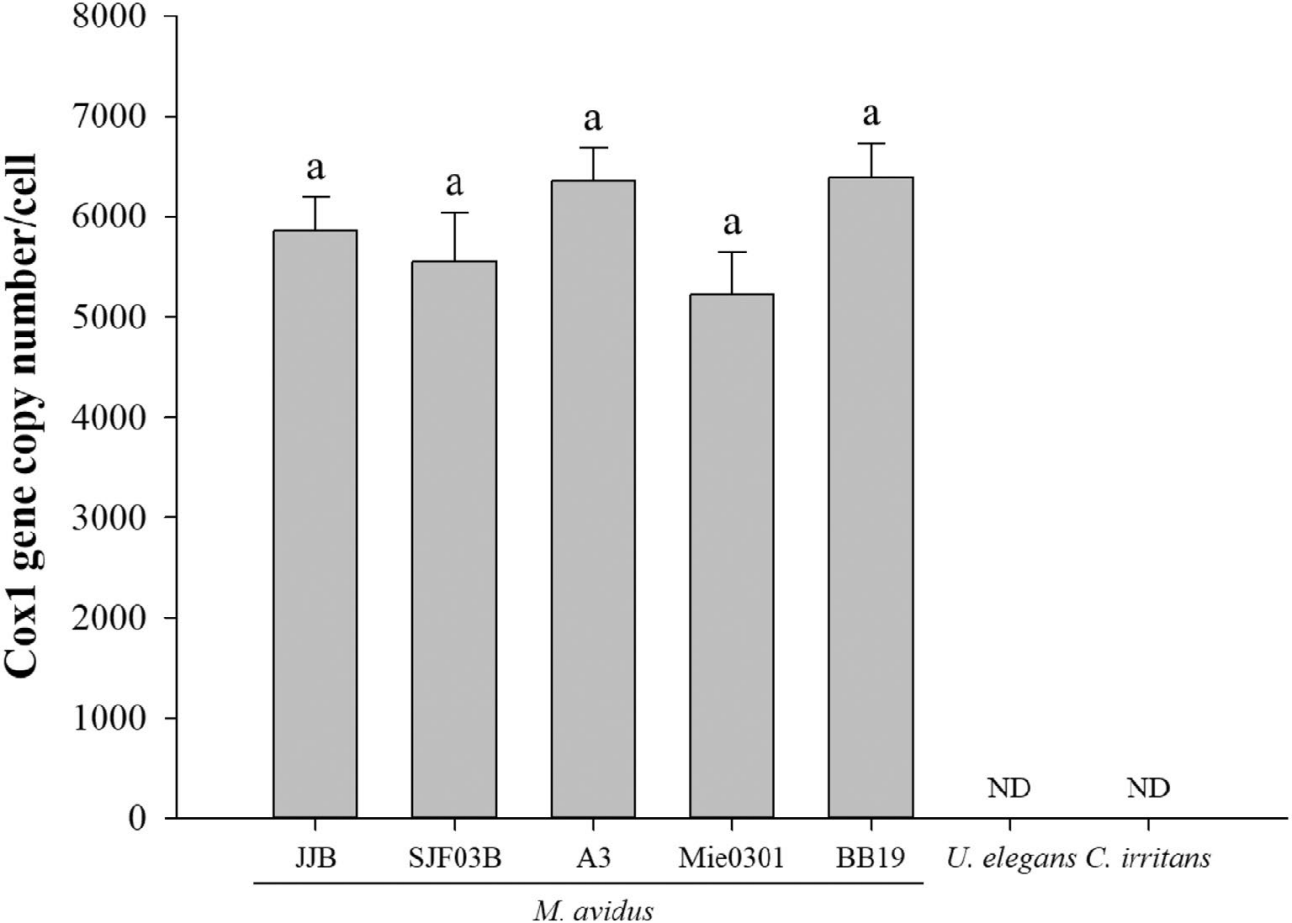
Specificity evaluation of the real‐time PCR assay against different *cox1* genotype strains of *M. avidus* (JJB: Genotype 1; SFJ‐03B: Genotype 2; A3: Genotype 3; Mie0301: Genotype 4; BB19: Genotype 4), *Uronema elegans* and *Cryptocaryon irritans*. ND: Not detected. Data are expressed as mean ± standard error of the mean (*n* = 3 per group). Different letters above the bars indicate significant differences between groups (*p* < 0.05).

Real‐time PCR targeting various *cox1* genotypes of *M*. *avidus* (five strains) successfully detected all genotypes. The *cox1* gene copy number per cell ranged from 4000 to 7000 across all strains, with the following estimates: JJB (*cox1* genotype 1), 5857 ± 585 copies/cell; SJF‐03B (*cox1* genotype 2), 5558 ± 833 copies/cell; A3 (*cox1* genotype 3), 6353 ± 540 copies/cell; Mie0301 (*cox1* genotype 4), 5220 ± 790 copies/cell; BB19 (*cox1* genotype 4), 6390 ± 592 copies/cell (Figure 3). There were no significant differences in copies per cell between the strains. Additionally, *U. elegans* and *C. irritans* were not detected in the real‐time PCR assay.

### 3.4 Sensitivity and Reproducibility Analysis

The assay could detect a single *M*. *avidus* cell in 1 L of seawater with a 70% probability and a 100% probability from a minimum of two cells or more (Table 3). *M*. *avidus* recovery rates ranged from 84.92% to 89.04% in all experimental groups. No *M*. *avidus* cells were detected in the 10 repeated experiments using natural seawater without *M*. *avidus*.

**TABLE 3:** Sensitivity and reproducibility analysis of *M. avidus* detection in seawater using the real‐time PCR assay.

Number of *M*. *avidus*	Ct value (mean ± SD)	Estimated *M*. *avidus* number/1 L (mean ± SD)	Recovery (%) (mean ± SD)	Positive signal samples	Detection rate (%)
0	35.88 ± 1.11	0.14 ± 0.10	0	0/10	0
1	32.94 ± 0.63	0.85 ± 0.30	85.03 ± 29.71	7/10	70
2	31.95 ± 0.78	1.74 ± 0.83	87.11 ± 41.50	10/10	100
4	30.78 ± 0.41	3.56 ± 1.07	89.04 ± 26.67	10/10	100
8	29.88 ± 0.53	6.79 ± 2.99	84.92 ± 37.27	10/10	100

### 3.5 Monitoring *M*. *Avidus*

#### 3.5.1 Detection From Fish and Rearing Water Across Developmental Stages

**FIGURE 4:**
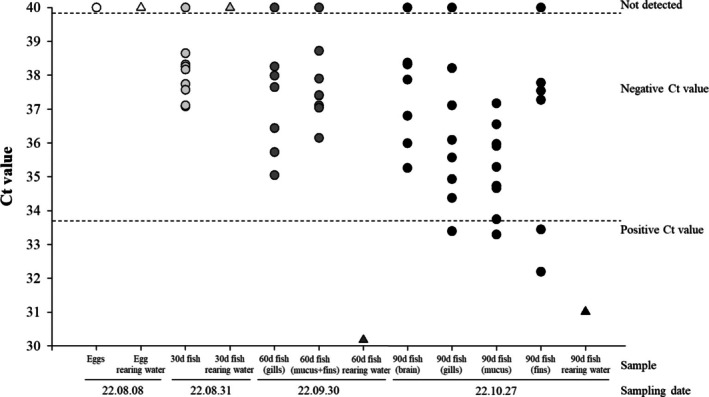
Comparison of detection sensitivity between invasive (200 μL egg samples, *n* = 2; fish samples, *n* = 10) and noninvasive (10‐L seawater samples, *n* = 1) methods for *M*. *avidus* in olive flounder and rearing water using real‐time PCR analysis. Data points are expressed as Ct values for individual samples (−●‐: Fish, −▲‐: Rearing water).

During the three production cycles, *M*. *avidus* was detected once during the second production cycle, from 8 August 2022, to 27 October 2022 (Figure 4). In this cycle, *M*. *avidus* was not detected in fertilised eggs, 30‐day‐old fish or in the rearing water from these stages. However, in the 60‐day‐old olive flounder samples, while *M. avidus* was absent in the fish, it was detected in the rearing seawater at an estimated concentration of 12.19 cells/10 L. In the 90‐day‐old olive flounder samples, collected on 27 October 2022, *M*. *avidus* was detected in both the fish and the culture water. Specifically, it was found in the gills, mucus and fins from one to two fish, even though they showed no clinical signs (Figure 4). Additionally, *M*. *avidus* was detected in the rearing seawater with an estimated concentration of 6.93 cells/10 L (Figure 4).

#### 3.5.2 Detection From Rearing Water in Diseased, Recovered and Healthy Olive Flounder Tanks

*M*. *avidus* was detected in the seawater from the tank experiencing an *M*. *avidus* infection, with an estimated concentration of 57.81 cells/10 L (Table 4). In contrast, *M*. *avidus* was not detected in the rearing seawater from the tank where the olive flounder had recovered from the infection, nor in the control tank with no history of *M*. *avidus* infection (Table 4).

**TABLE 4:** Detection of *M. avidus* in the rearing water of diseased, recovered and healthy olive flounder.

Date	Water temperature	Group	Ct value	Cox1 copy number/1 μL DNA	Estimated *M*. *avidus* number/10 L
22.08.10	23.7°C	Diseased	27.89	1737.31	57.81
		Recovered	35.04	—	—
		Control	35.91	—	—

#### 3.5.3 Detection From Inflow Natural Seawater Into Farm and Effluent Rearing Water

During the year‐long monitoring of seawater at the olive flounder farm, *M*. *avidus* was detected in the inflow seawater on 27 October 2022, and 21 March 2023 (Table 5). Additionally, *M*. *avidus* was detected in the effluent rearing water on five occasions: March 23, 2022, August 31, 2022, January 12, 2023, February 20, 2023 and April 20, 2023 (Table 5).

**TABLE 5:** Detection of *M. avidus* in inflow natural seawater entering the olive flounder farm and in effluent rearing seawater collected from the sedimentation tank before discharge into the sea using a real‐time PCR assay.

	Inflow seawater		Effluent rearing seawater	
Date	Ct value	Estimated *M*. *avidus* number/10 L	Ct value	Estimated *M*. *avidus* number/10 L
22.03.23	35.14	—	27.35	83.30
22.04.27	ND	—	36.72	—
22.05.23	36.28	—	ND	—
22.06.22	35.03	—	37.10	—
22.07.21	35.93	—	36.60	—
22.08.08	ND	—	ND	—
22.08.31	37.00	—	32.82	2.02
22.09.30	36.59	—	38.04	—
22.10.27	30.76	8.22	35.59	—
22.11.29	35.06	—	35.37	—
22.12.28	36.05	—	35.29	—
23.01.12	36.96	—	32.09	3.32
23.02.20	ND	—	33.00	1.79
23.03.21	33.26	1.49	ND	—
23.04.20	ND	—	32.78	2.07

Abbreviation: ND, not detected.

## 4 Discussion

In real‐time PCR analysis, careful selection of the target gene is crucial for enhancing the sensitivity and specificity of pathogen detection. The mitochondrial *cox1* gene has been widely used as a reliable marker for microbial identification and detection (Budiño et al. 2011; Guangxun et al. 2019; Song et al. 2022). This gene is highly suitable for this purpose due to its high copy number and significant sequence variability within mitochondria (Jung et al. 2011b; Whang et al. 2013). Studies have shown that protozoan parasites such as *Cytauxzoon felis* and *Toxoplasma gondii* are more sensitively detected by targeting the cytochrome c oxidase subunit gene than the 18S rRNA gene (Feng et al. 2017; Schreeg et al. 2016). Therefore, in this study, the mitochondrial *cox1* gene was selected as the target for developing real‐time PCR for *M*. *avidus*.

Amplification efficiency, which is derived from the slope of the standard curve through linear regression, is an important factor in determining the accuracy of real‐time PCR (Rao et al. 2013). In this experiment, the *cox1* gene standard curve showed a slope of −3.4146 with excellent linearity, corresponding to an amplification efficiency of 96.26%. This value falls within the acceptable efficiency range of 90%–110% (Taylor et al. 2019), indicating high accuracy in detecting *M*. *avidus*.

While gene copy numbers can be quantified, the number of parasites is ultimately more indicative of the disease severity. To the best of our knowledge, there is limited information on mitochondrial DNA amounts in ciliates. In this study, we quantified the *cox1* gene copy number in *M*. *avidus* mitochondria and found it to be 6017 copies per cell. This is approximately 366 times fewer than the 2.2 million copies of the mitochondrial cytochrome b gene (cytb) found in the trematode *Neobenedenia girellae* (Agawa et al. 2016), a much larger parasite (3.7–4.9 mm vs. 21–37 μm for *M*. *avidus*). These differences in mitochondrial genome number may be attributed to variations in parasite species and size. Based on this *cox1* gene copy number, we were able to estimate the number of *M*. *avidus* cells in unknown samples.

Our real‐time PCR was able to specifically detect *M. avidus* among various organisms in natural seawater and did not detect *Uronema elegans* or *Cryptocaryon irritans*, ciliates that may be present in olive flounder farms. Since our assay consistently detected only *M. avidus* cox1 genotypes 1–4 (five strains), it demonstrates high specificity for *M. avidus*.

The detection limit for positive plasmids was approximately 20 copies/μL, theoretically equivalent to 0.3 *M*. *avidus* cells. However, this detection limit may be influenced by factors such as the seawater filtration process and DNA extraction efficiency. To evaluate the efficiency of *M*. *avidus* DNA recovery from seawater, we conducted sensitivity and reproducibility tests. *M*. *avidus* cells were spiked into 1 L of seawater at concentrations of 0, 1, 2, 4 and 8 cells, and their DNA was detected following our protocol. We successfully detected a single *M*. *avidus* cell from 1 L of seawater in 7 out of 10 replicates. Additionally, detection was successful with 100% probability when at least two *M*. *avidus* cells were present in 1 L of seawater across all 10 replicates. The recovery rate averaged over 80%, which is consistent with the generally accepted recovery range of 80%–120% (Sommers et al. 2018). Therefore, our method enables reliable detection of *M*. *avidus* with minimal sample loss and high reproducibility, even at low concentrations.

The sensitivity of invasive and noninvasive methods for detecting *M*. *avidus* was compared in olive flounder tissues and fish rearing water samples as the fish grew from fertilised eggs to 90 days old, marking the transfer period from hatchery to culture farm. Detection of *M*. *avidus* was conducted monthly over three production cycles for 1 year. Notably, *M*. *avidus* was detected only once during this period, as shown in Figure 4. *M*. *avidus* was identified in the rearing seawater at 60 days of age, approximately 1 month before it was detected in olive flounder at 90 days. Furthermore, noninvasive methods utilising rearing water samples demonstrated greater sensitivity compared to invasive techniques requiring the sacrifice of olive flounder. The noninvasive method developed in this study offers advantages for earlier and more sensitive detection, enabling timely treatment when pathogen loads remain low.

Noninvasive sampling offers advantages for disease surveillance. The number of samples required for disease surveillance in aquatic animal populations depends on the expected prevalence (threshold prevalence), confidence level and the sensitivity and specificity of the diagnostic test. For example, with 100% sensitivity and specificity, 149 samples are needed at a 2% prevalence, and 59 samples are required at a 5% prevalence (World Organisation for Animal Health 2024). This underscores the challenge of relying solely on invasive methods, which require sacrificing a significant number of fish for effective disease surveillance. In contrast, the findings of this study suggest a more practical and cost‐effective alternative for routine screening before clinical signs appear. By using noninvasive sampling of rearing water, pathogen detection can be conducted without handling or sacrificing fish, reducing labour costs, stress‐related mortality and sampling effort. Additionally, this approach is well suited for large‐scale field monitoring, as water samples can be easily collected and tested using PCR without requiring specialised expertise in fish handling or dissection. Therefore, integrating this method into routine surveillance programmes could significantly enhance the efficiency and feasibility of disease monitoring in aquaculture.

The implementation of this assay in fish farms may face challenges such as the need for specialised equipment, trained personnel and logistical considerations for sample collection and processing. Setting up on‐site PCR testing in individual fish farms may not always be practical due to these constraints. However, as conducted in our study, a feasible alternative is to collect water samples from fish farms and send them to local disease inspection centres or university laboratories for analysis. In particular, aquatic animal health inspectors can collect water samples from multiple fish farms and submit them to a centralised testing facility. This approach could enable efficient, large‐scale disease surveillance within a short period. This centralised testing model would reduce the burden on individual farms while ensuring accurate and timely detection of *M. avidus* infections, ultimately supporting better disease management in aquaculture systems.

Next, we aimed to quantify the level of *M*. *avidus* present in the rearing water of olive flounder exhibiting clinical signs of infection. In a tank with infected olive flounder, a concentration of 57.81 cells/10 L was detected, which is considered highly threatening and is associated with increased mortality rates in aquaculture tanks. Although olive flounder may appear asymptomatic after recovering from infection, the parasite can persist in their bodies, particularly in the brain, and may re‐emerge after a period of being clinically unnoticeable (Kim et al. 2019; Tange et al. 2010). To investigate the effectiveness of our noninvasive methods in detecting *M*. *avidus* in recovered olive flounder, we aimed to assess whether this approach could identify cases where the parasite persists in the brain without manifesting clinical signs on the body's surface. However, *M*. *avidus* was not detected in the recovered olive flounder. Since the extent of persistent infection in the fish population was not evaluated in this study, additional follow‐up observations on clinical signs and parasite loads in rearing water may be necessary. Further investigations should focus on the long‐term monitoring of parasite persistence, particularly in asymptomatic fish, as well as on environmental factors such as water quality and stress levels that could trigger a recurrence of the infection. In parallel, to minimise the potential transmission of *M. avidus* from tanks containing persistently infected fish to healthy fish, strict biosecurity measures should be implemented. These include maintaining physical separation between tanks, using dedicated equipment for each tank, and preventing cross‐contamination of water sources. Additionally, proper disinfection of equipment and personnel is essential to further reduce the risk of pathogen spread. Integrating long‐term monitoring with robust biosecurity measures can provide a more comprehensive understanding of *M. avidus* infection dynamics in aquaculture systems, ultimately improving prevention and management strategies.

During a 1‐year monitoring period, *M*. *avidus* was detected in the inflow seawater to the olive flounder farm in two out of 15 tests. This finding underscores the importance of managing the introduction of the parasite through inflow water for successful seed production. Although the hatchery in this study employs electrolysis and UV disinfection for inflow water before its use in seed production, our results indicate that completely preventing the entry of the parasite remains a challenge. Electrolysis and UV disinfection significantly reduce pathogen levels but cannot ensure complete elimination due to the large volume of seawater used, which exceeds the capacity of these disinfection systems—particularly during abnormal events such as typhoons or strong winds that increase water turbidity, further limiting their ability to provide sufficient disinfection. In addition, *M*. *avidus* was detected five times out of 15 tests in the effluent water, demonstrating a higher detection frequency and DNA quantity compared to the inflow water. Notably, in March 2022, the total discharge water exhibited a Ct value of 27.35, which is similar to that observed in infected tanks, indicating the presence of approximately 83 *M*. *avidus* cells per 10 L of water. These findings highlight the critical need for more stringent biosecurity measures, including regular monitoring of both inflow and discharge water, as well as the implementation of advanced filtration and disinfection technologies to mitigate the risk of pathogen introduction and transmission in aquaculture systems.

In conclusion, the real‐time PCR assay developed in this study provides a fast, convenient and accurate method for detecting and quantifying *M*. *avidus* in rearing seawater samples without causing fish sacrifice or stress. This assay instils high confidence in determining the presence of *M*. *avidus* in olive flounder farms, enabling the implementation of more effective prevention and response measures against the disease.

## Author Contributions

Jin‐Young Kim: conceptualisation, methodology, writing – original draft, investigation, software, visualisation. Su‐Mi Shin: investigation. Se Ryun Kwon: writing – review and editing. Sung‐Ju Jung: conceptualisation, writing – review and editing, supervision, funding acquisition.

## Conflicts of Interest

The authors declare no conflicts of interest.

## Supporting information


**Table S1.** List of sequences used to align the cox1 gene of ciliates for designing real‐time PCR primers for *M*. *avidus*.

## Data Availability

The data that support the findings of this study are available from the corresponding author upon reasonable request.
