# Supplementary material for: Development of a Real‐Time PCR Assay for Noninvasive Detection of Miamiensis avidus in Olive Flounder Aquaculture
Source: J Fish Dis. 2025 Apr 23;48(10):e14134. doi: 10.1111/jfd.14134 (PMC12421793; doi:10.1111/jfd.14134)
Supplement: Supplementary file 1 — Table S1. List of sequences used to align the cox1 gene of ciliates for designing real‐time PCR primers for M. avidus. [file JFD-48-e14134-s001.docx]

**Supplementary data**

**Table S1.** List of sequences used to align the cox1 gene of ciliates for designing real-time PCR primers for *M*. *avidus*.

| **Species** | **Isolate** | **Genbank accession number** |
| --- | --- | --- |
| *Miamiensis avidus* | WS1 | EU831225.1 |
| *Miamiensis avidus* | JJ4 | EU831219.1 |
| *Miamiensis avidus* | Nakajima | EU831226.1 |
| *Miamiensis avidus* | SJF-03B | EU831216.1 |
| *Miamiensis avidus* | SK05Kyo | EU831232.1 |
| *Miamiensis avidus* | YK2 | EU831229.1 |
| *Miamiensis avidus* | A3 | EU831214.1 |
| *Miamiensis avidus* | Mie0301 | EU831233.1 |
| *Miamiensis avidus* | YS3 | EU831218.1 |
| *Miamiensis avidus* | 2017SHK-LS11 | MH078246.1 |
| *Miamiensis avidus* | 2017SHK-LS12 | MH078247.1 |
| *Miamiensis avidus* | xiapu1 | MN688231.1 |
| *Miamiensis avidus* | shaceng1 | MN688232.1 |
| *Philasterides dicentrarchi* | - | MN531306.1 |
| *Uronema marinum* | ZZF2017040 | MG001903.1 |
| *Uronema marinum* | PHB090219 | MH605541.1 |
| *Uronema heteromarinum* | FXP08082901 | MH605535.1 |
| *Uronema heteromarinum* | FXP08082901 | MH605535.1 |
| *Pseudocohnilembus persalinus* | - | GU584095.1 |
| *Pseudocohnilembus hargisi* | JJM2010031301 | MH605559.1 |
| *Trichodina baltica* | KR_115_TF | ON968466.1 |
| *Trichodina unionis* | SL_55_UT | ON968478.1 |
| *Trichodina unionis* | HO_15_UT | ON968471.1 |
| *Metanophrys sinensis* | FXP09052901 | MH605565.1 |
| *Metanophrys orientalis* | PXM10101302 | MH605551.1 |
| *Mesanophrys carcini* | PXM10022606 | MH605547.1 |
| *Tetrahymena borealis* | 20105-1 | KY217972.1 |
| *Tetrahymena thermophila* | ANF1359-1 | DQ411879.1 |
| *Tetrahymena rostrata* | ID-3 | DQ411883.1 |
| *Tetrahymena elliotti* | MP80 | EF070281.1 |
| *Tetrahymena canadensis* | 19552-1 | KY218008.1 |
| *Tetrahymena thermophila* | A 1298-7a | DQ411877.1 |
| *Tetrahymena hyperangularis* | EN112 | DQ411880.1 |
| *Tetrahymena hegewischi* | 19262-1 | Y218128.1 |
| *Tetrahymena tropicalis* | 20922-1 | KY218496.1 |
| *Ichthyophthirius multifiliis* | TW5 | KT783597.1 |
| *Ichthyophthirius multifiliis* | G15 | KJ690548.1 |
| *Ichthyophthirius multifiliis* | ImulTR3 | MN687777.1 |
| *Cryptocaryon irritans* | JP_TY_86 | KY427004.1 |
| *Cryptocaryon irritans* | UAE-AD-2022 | PQ287301.1 |
